# Simultaneous Aortic Dissection and Saddle Pulmonary Embolism: Were They Intertwined?

**DOI:** 10.7759/cureus.98377

**Published:** 2025-12-03

**Authors:** Miguel João Amaral de Vasconcelos Pinheiro, Tiago Serrano Constantino, João Fonseca Oliveira, Ana Maria Cordeiro, Sofia Pinheiro

**Affiliations:** 1 Internal Medicine, Hospital São José, Hospital Santo António dos Capuchos, Lisbon, PRT; 2 Cardiology Service, Hospital Santa Marta, Lisbon, PRT; 3 Internal Medicine, Centro Hospitalar Universitário de Lisboa Central, Lisbon, PRT; 4 Internal Medicine, Nova Medical School, Lisbon, PRT; 5 Radiology, Hospital Santo António dos Capuchos, Centro Hospitalar Universitário Lisboa Central, Lisbon, PRT; 6 Medicine Service, Hospital Santo António dos Capuchos, Lisbon, PRT

**Keywords:** aortic thrombosis, complications of aortic dissection, medular infarct, saddle pulmonary embolism, stanford type b dissection, symptoms of aortic dissection, therapeutic anticoagulation

## Abstract

We report the case of a 92-year-old woman who developed sudden paraplegia. A magnetic resonance imaging (MRI) of the dorsal spine revealed extensive spinal cord infarction. Subsequent imaging demonstrated a Stanford type B aortic dissection complicated by false-lumen thrombosis, along with a coexistent saddle pulmonary embolism. Although the coexistence of pulmonary embolism and aortic dissection has been reported, it remains poorly understood and represents a major therapeutic challenge. In this case, the close anatomical relationship between the descending aortic dissection and the site of pulmonary artery thrombosis strongly suggests that the aortic dissection may have contributed to pulmonary thrombus formation through direct mechanical compression and disturbed local hemodynamics arising from their anatomical contiguity. This case illustrates a rare yet clinically significant overlap between two life-threatening vascular entities, suggesting a poorly characterized pathophysiological interplay and underscoring the need for early diagnosis and patient-specific therapeutic approaches.

## Introduction

Acute aortic dissection is a life-threatening medical emergency with a high mortality rate. It occurs when an injury to the innermost layer of the aortic wall allows blood to enter and separate the layers of the vessel. The major risk factors include hypertension, connective tissue disorders, a congenitally bicuspid aortic valve, and aortic coarctation [1,2].

Patients with aortic dissection may present with a broad spectrum of symptoms; therefore, diagnosis requires a high index of clinical suspicion. Chest pain is the most frequent presenting symptom, but pulse deficits or a new aortic regurgitation murmur may also be observed. Anterior chest pain is more typical in dissections involving the ascending aorta, whereas back or abdominal pain predominates in dissections of the descending aorta [2].

The Stanford classification of aortic dissections is clinically useful, as it aligns with therapeutic strategies: type A dissections, involving the ascending aorta, generally require urgent surgical repair, whereas type B dissections, characterized by an intimal tear originating distal to the left subclavian artery, are usually managed medically [3]. Nonetheless, in Stanford type B aortic dissection, patients who are hemodynamically unstable or present with critical organ malperfusion generally require urgent surgical or endovascular intervention. Patients with uncomplicated Stanford type B dissection are typically managed medically, focusing on strict control of blood pressure, heart rate, and modifiable cardiovascular risk factors [3]. Regular surveillance with periodic imaging and clinical reassessment is essential to detect disease progression or complications [4].

From a pathophysiological perspective, if left untreated, aortic dissection may lead to progressive aortic enlargement, aneurysm formation, late rupture, and new or recurrent dissection. This may result in severe complications, such as spinal cord ischemia, either from direct extension of the dissection flap obstructing critical radicular arteries or from systemic hypoperfusion due to compromised aortic blood flow.

Thrombosis of the false lumen may develop when stagnant flow and endothelial injury within the dissected aortic wall activate coagulation pathways. Partial thrombosis of the false lumen is defined as the concurrent presence of both flow and thrombus within the false lumen. In particular, complete false-lumen thrombosis has been associated with more favorable outcomes, as it is less likely to progress to aortic dilation or rupture owing to reduced pressurization within the false channel [5].

In addition, pulmonary embolism (PE) is another life-threatening vascular condition that shares several risk factors with aortic dissection, including advanced age, hypertension, and atherosclerosis [6]. Although both diseases are well characterized individually, the coexistence of PE and aortic dissection is rare, especially in Stanford type B aortic dissection, and the relationship between them remains insufficiently studied [7]. Several hypotheses may be proposed to explain this association, including coincidental occurrence in patients with shared risk profiles or a direct mechanical compression of the pulmonary arteries by the dissected aorta, which may predispose to in situ pulmonary thrombosis. Consequently, the overlap of these two medical emergencies poses a major therapeutic challenge, as systemic anticoagulation, the cornerstone of PE management, may precipitate rupture in the setting of aortic dissection.

## Case presentation

This case reports a 92-year-old woman with a previous history of epilepsy, well-controlled on phenytoin, and without documented cardiovascular risk factors, such as hypertension, dyslipidemia, or tobacco use, who presented to the emergency department with one month of fatigue and exertional dyspnea. She exhibited no significant cognitive or functional impairment. At that time, new-onset atrial fibrillation was diagnosed, and the patient was discharged with apixaban and bisoprolol.

Three days later, the patient returned to the same hospital with a sudden onset of paraplegia, loss of pain and temperature sensation below the T7 level, accompanied by urinary retention. The patient was hemodynamically stable and eupneic on room air. Laboratory blood tests obtained on admission showed elevated NT-proBNP and C-reactive protein levels with normal troponin, and no other significant abnormalities, as shown in Table 1.

**Table 1:** Summary of the laboratory results on admission

Variable	Result	Units
White blood cell count (WBC)	10.09	x 10^9^/L
C-reactive protein (CPR)	83	mg/L
Hemoglobin (Hb)	11.8	g/dL
Platelets	240.000	x 10^9^/L
NT-proBNP	3509	pg/mL
Troponin	14.7	ng/L
Urea	36	mg/dL
Creatinine	1.26	mg/dL
Aspartate aminotransferase (AST)	22	U/L
Alanine aminotransferase (ALT)	21	U/L
Gamma-glutamyl transferase (GGT)	129	U/L
Alkaline phosphatase (ALP)	118	U/L

**Figure 1:**
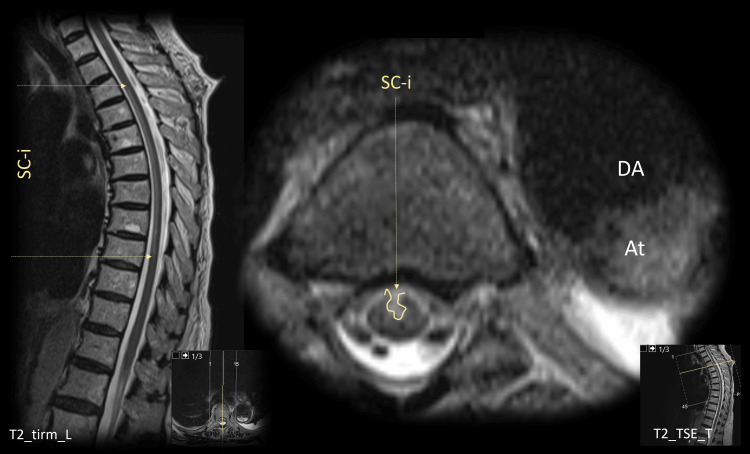
Spinal cord MRI At - Aortic thrombosis; DA - Descending Aorta; SC-i - Spinal Cord Infarction

Due to the neurological deficits, an urgent thoracic MRI scan was performed and revealed an extensive spinal cord infarction, extending from T3 to T9 level (Figure 1). This lesion was compatible with the neurological deficits of the patient.

**Figure 2:**
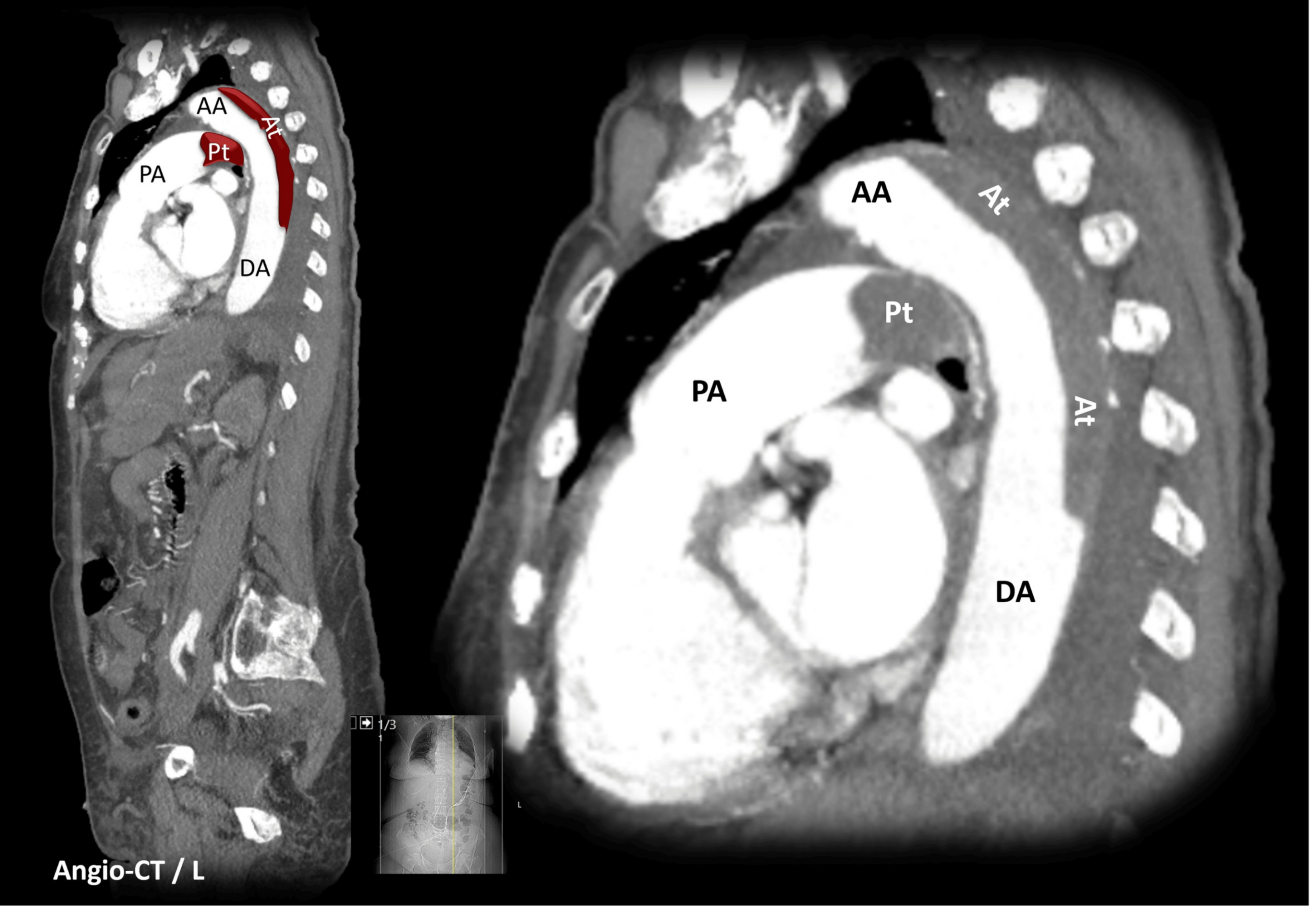
Chest angio computed tomography (longitudinal reformatting) AA - Ascending Aorta; At - Aortic Thrombosis; DA - Descending Aorta; PA - Pulmonary Artery; Pt - Pulmonary Thrombosis

**Figure 3:**
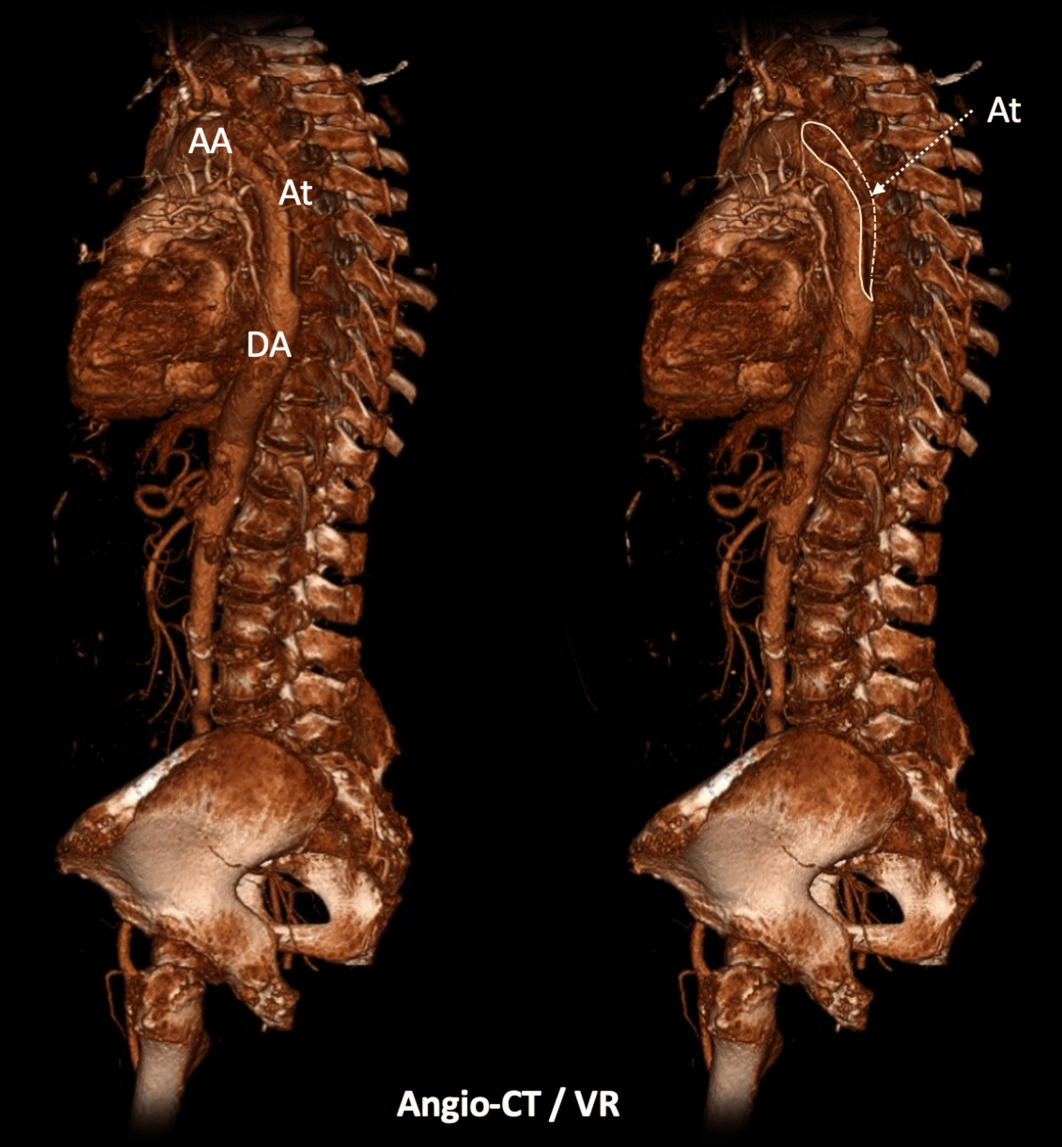
Angio computed tomography - volume rendering AA -Ascending Aorta; At - Aortic Thrombosis; DA - Descending Aorta

Additionally, this thoracic MRI scan immediately raised suspicion for an underlying aortic dissection. Therefore, a thoracic, abdominal, and pelvic computed tomography (CT) was promptly performed, and confirmed the diagnosis of a Stanford type B aortic dissection with mural thrombosis, beginning at the distal portion of the aortic arch and continuing throughout the descending aorta (Figures 2-3). 

**Figure 4:**
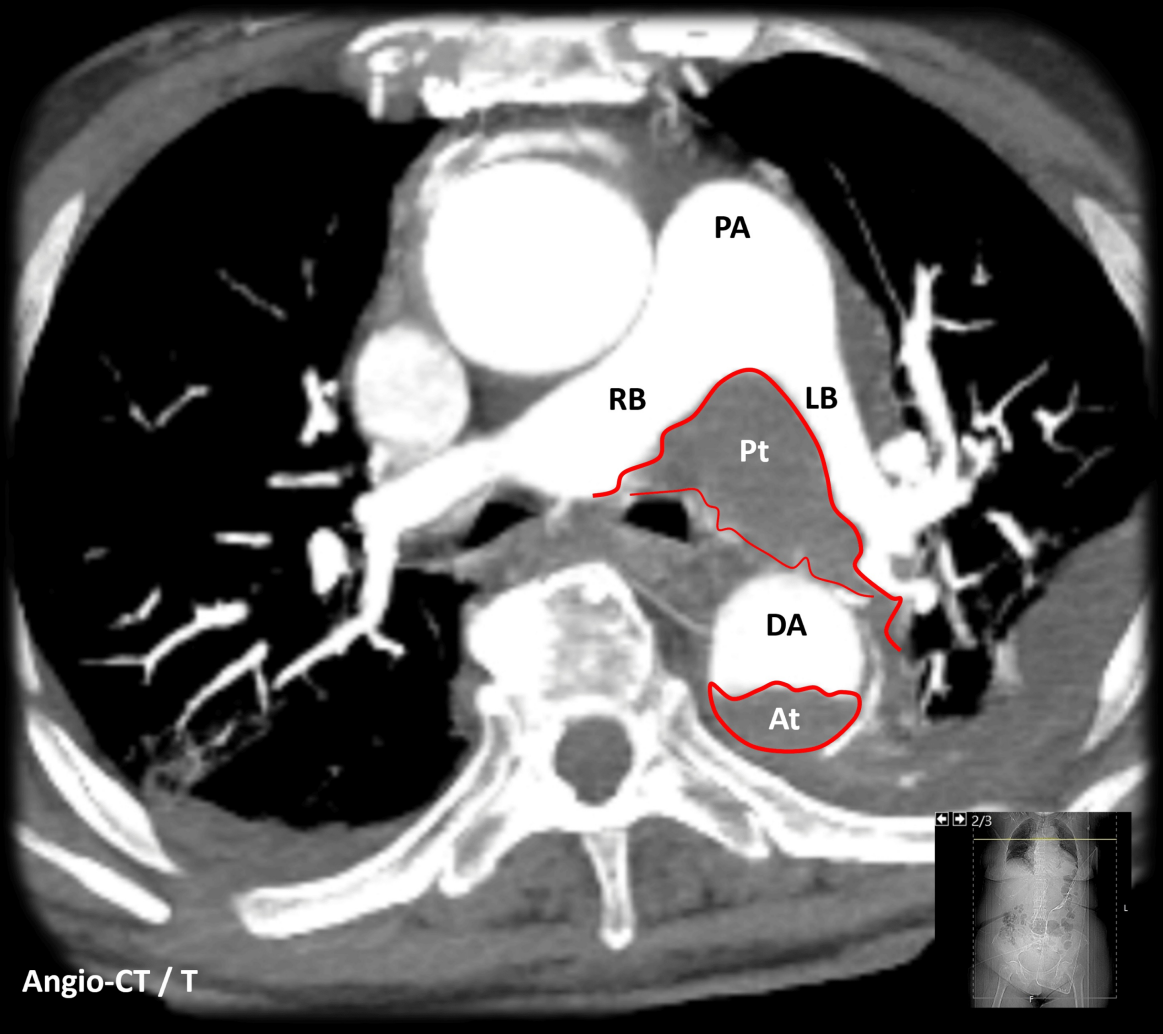
Chest angio computed tomography (transversal reconstruction) At - Aortic Thrombosis; DA - Descending Aorta; LB - Left Branch; PA - Pulmonary Artery; Pt - Pulmonary Thrombosis; RB - Right Branch

The CT scan also revealed a large saddle pulmonary embolism, a thrombus that straddles the bifurcation of the main pulmonary artery and both main bilateral branches. Furthermore, it showed a direct complication of the pulmonary embolism: dilation and opacification of the hepatic veins, findings consistent with right ventricular overload (Figure 4). Unfortunately, an echocardiogram was not performed. Additionally, the CT scan noted reduced enhancement of the anterior aspect of the left kidney, which was interpreted as renal infarction due to hypoperfusion.

The patient was hospitalized for clinical surveillance. Subsequently, given the submassive pulmonary embolism, defined by the stable blood pressure at admission, no myocardial necrosis, and evidence of right ventricular dysfunction, seen on the thoracic CT scan and elevated NT-proBNP, anticoagulation was initiated despite the known hemorrhagic risk associated with aortic dissection. As the patient was hospitalized, anticoagulation was achieved with therapeutic doses of enoxaparin at a dose of 1 mg/kg subcutaneously every 12 hours. This anticoagulant was selected in part due to its shorter half-life compared to other oral anticoagulants.

Regarding the Stanford type B aortic dissection, initial hemodynamic stability permitted conservative management, with heart-rate control achieved using bisoprolol. Antihypertensive drugs were not needed. Unfortunately, the dissection progressed over the following days, resulting in refractory hypotension and systemic tissue hypoperfusion, specifically with left arm ischemia, and ultimately cardiac arrest.

## Discussion

Although rare, the presentation of aortic dissection with associated spinal cord infarction is well documented in the literature. The most common clinical manifestation of spinal cord infarction is anterior spinal artery syndrome, occurring in up to 87% of cases [8].

On the other hand, the association between aortic dissection and pulmonary embolism is infrequent. In the case of this patient, there was an imperative need to treat a submassive pulmonary embolism with anticoagulation, but this option may have contributed to the recanalization of the aortic thrombosis with increased false-lumen patency, leading to aortic dissection complications.

Some authors suggest that patients with acute aortic dissection exhibit a hypercoagulable state, resulting in a high risk of venous thromboembolism in the acute phase [9]. In addition, in this specific case here reported, besides advanced age, which is a common risk factor for both pathologies, one could argue that the immobility due to acute paraplegia may have further predisposed this patient to deep vein thrombosis and subsequent pulmonary embolism. Nevertheless, these facts alone might be insufficient to explain this complex coexistence.

In fact, previous case reports have described the compression of the pulmonary artery by a chronic ascending aortic aneurysm leading to pulmonary hypertension [10] and acute dissecting aneurysm of the ascending thoracic aorta causing obstruction and thrombosis of the pulmonary arteries [11]. Thus, it is reasonable to consider that hemodynamic alterations of the descending aorta may also interfere with the adjacent pulmonary vasculature. In this case, the anatomical proximity between the descending aortic dissection and the saddle pulmonary embolism raises the hypothesis that mechanical compression of the pulmonary artery by the dissected aorta could lead to pulmonary blood flow stagnation, endothelial lesion, and thrombus formation.

However, this underlying mechanism linking type B aortic dissection and pulmonary embolism remains unclear; therefore, further studies are warranted to elucidate this association. Studies integrating advanced imaging and patient-specific computational fluid dynamics are needed to determine how anatomical features, flow disturbances, and mechanical compression in aortic dissection may contribute to pulmonary embolism risk and pathophysiology.

## Conclusions

The association of PE and aortic dissection presents a therapeutic dilemma and can be particularly challenging to manage. Although anticoagulation is the cornerstone of treatment for PE, it carries a significant risk of hemorrhage in the setting of aortic dissection, potentially leading to cardiac tamponade or aortic rupture.

This case highlights the complex relationship between these vascular conditions and presents imaging findings that strongly suggest that the anatomical contiguity of the aortic dissection and the pulmonary artery directly contributed to the concurrent saddle PE. Therefore, it should raise awareness of this potential complication of Stanford type B dissection. This report also underscores a gap in the current literature by suggesting that aortic dissection may exert hemodynamic and structural effects on the surrounding vascular structures, an interaction that remains poorly characterized. Further studies, including advanced computational fluid dynamics analysis, are needed.
